# Solid Lipid Nanoparticles as Carriers of Natural Phenolic Compounds

**DOI:** 10.3390/antiox9100998

**Published:** 2020-10-15

**Authors:** Alexandra Borges, Victor de Freitas, Nuno Mateus, Iva Fernandes, Joana Oliveira

**Affiliations:** LAQV-REQUIMTE, Departamento de Química e Bioquímica, Faculdade de Ciências da Universidade do Porto, Rua do Campo Alegre, S/N, 4169-007 Porto, Portugal; up201503108@fc.up.pt (A.B.); vfreitas@fc.up.pt (V.d.F.); nbmateus@fc.up.pt (N.M.)

**Keywords:** solid lipid nanoparticles, nanostructured lipid carriers, phenolic compounds, bioactivity, chemical stabilization

## Abstract

Phenolic compounds are one of the most widespread classes of compounds in nature, with several beneficial biological effects being associated with their anti-oxidant and anti-carcinogenic activities. Their application in the prevention or treatment of numerous chronic diseases have been studied, but a major drawback is still the low bioavailability of these compounds, as well as their instability towards pH, temperature, and light in some cases. Nanotechnology has emerged as an alternative to overcome these limitations, and the use of lipidic encapsulation systems is a promising technique to achieve an efficient drug delivery, protecting molecules from external factors and improving their bioavailability. In this review, solid lipid nanoparticles and nanostructured lipid carriers are highlighted as an important tool for the improvement of the bioavailability and stability of natural phenolic compounds, including their preparation methods and functionalization approaches and the discussion of several applications for putative use in cosmetic and pharmacologic products.

## 1. Introduction

**Figure 1:**
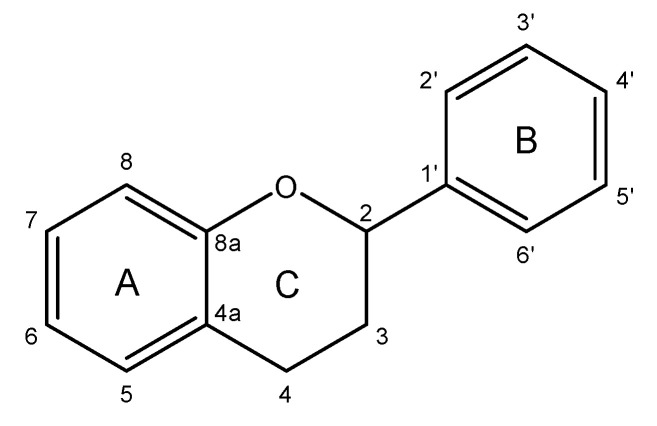
Structure of the flavanic core. A and B aromatic rings and C pyranic ring.

Phenolic compounds are natural compounds synthesized by plants that play an important role in cellular growth, coloration, and regulation of fruits’ maturation, being present in many vegetal foodstuffs, flowers, and beverages [1,2]. From a structural point of view, these molecules are characterized by an aromatic ring with one or more hydroxyl groups and can be classified by considering the number of phenol rings and their substitution pattern. Consequently, phenolic compounds can be categorized as non-flavonoids, which include phenolic acids, stilbenes and lignans, or flavonoids, which share a common structure, the flavanic core, consisting of 15 carbon atoms arranged in three rings, A, B, and C (Figure 1), and includes flavan-3-ols, flavonols, flavanones, anthocyanins, flavones, and isoflavones.

These compounds have been postulated as anti-oxidants, anti-mutagens, chelators of catalytic metals, and free radical scavengers [3]. Moreover, the activity of these molecules against some diseases has been described, with such effects not only being linked to the antioxidant properties, but also with a more complex mechanism involving the cell signaling pathway [4]. The great abundance of phenolic compounds in the diet combined with their notorious role in the prevention of several health conditions turned these compounds into a substantial target for nutritionists, food scientists, and even drug and cosmetic developers (Table 1). This is particularly important as functional foods and nutraceuticals are rapidly being integrated into the industrial mainstream and are being increasingly accepted by the consumers due to a growing demand for natural and healthier products [5]. It is important to note that the beneficial properties of phenolic compounds observed in in vitro experiments are not verified in the same extent in vivo, since there are some drawbacks associated with these metabolites, for instance their bioavailability and their instability to some external factors such as light, temperature, and oxygen. Thus, it is expectable that the incorporation of phenolic compounds in food or cosmetic matrices can be a challenge due to the processing techniques and storage that are likely to compromise molecules’ integrity. Moreover, the application in foods of some phenolic compounds might affect directly flavor and color, or indirectly due to interactions between these compounds and other food components. On the other hand, phenolic compounds might also affect food nutritional value since they can interact (form a complex) with proteins and carbohydrates, making them inaccessible for absorption [6,7]. Polyphenols are described as interacting with digestive enzymes, inhibiting their activity and consequently reducing nutrient absorption [8].

The low values of bioactivity observed for polyphenols in the human body can be attributed either to a lower intrinsic activity, a poor absorption at the intestine level, or a high metabolization process [2]. On the other hand, some evidence points out that effects observed for some phenolic compounds in vivo are not derived from the ingested molecules but from their metabolites [9]. It is undoubtedly the case that the effectiveness of phenolic compounds depends on preserving their stability, bioactivity, and possibly enhancing their bioavailability, which can be achieved with encapsulation approaches.

The use of encapsulated phenolic compounds rather than free compounds has been explored over time to overcome the mentioned issues and to ensure an adequate delivery of the molecule into the bloodstream. Encapsulation consists of loading a compound into a capsule, which can be seen as a sphere [10]. A major advantage of this process is that usually the external phase or coating can be changed according to the main goal, thereby influencing parameters such as the release of the encapsulated compound, size, or affinity of capsules for the final material. Over the years, there has been a growing interest in nano-range materials (2–1000 nm) because these particles may be used as drug/active compound carriers. Synthetic and natural polymeric nanoparticles have been explored, as well as other types of nanocarriers such as fullerenes and carbon nanotubes. However, there are some relevant problems related to the use of nanoparticles such as their cytotoxicity and/or the difficulty of scaling up the production process [11].

In 1991, Muller developed a revolutionary way to overcome various limitations associated with other encapsulation methods by introducing solid lipid nanoparticles (SLNs) [26]. SLNs have the particularity of being constituted by lipids that remain solid at both the temperature of the human body and at room temperature, being of great use when the main goal is to achieve a controlled drug release while developing formulations, as will be further explored. Furthermore, their particle size usually varies from 40 to 1000 nm, which provides a larger offer of nanoparticle possibilities according to special needs [27]. SLNs present several advantages over other colloidal carriers such as higher drug stability, incorporation of both hydrophilic and lipophilic drugs, absence of toxicity, easy large-scale production, and the possibility of lyophilization [11]. More recently, a second generation of lipid nanoparticles emerged—the nanostructured lipid carriers (NLCs), which are made of both solid and liquid lipids. The composition of these nanoparticles, along with their characteristics, make SLNs and NLCs ideal for the carrying/delivery of sensitive bioactive compounds, protecting them against chemical degradation and also facilitating their application in varied administration routes. The successful loading of phenolic compounds such as resveratrol and quercetin into SLNs or NLCs have been reported in numerous experiments with promising results [28,29].

Bearing this in mind, this review focuses on comparison between SLN and NLC nanocarriers, including the preparation methods and functionalization approaches, providing an insight on their importance in the encapsulation of phenolic compounds.

## 2. Solid Lipid Nanoparticles and Nanostructured Lipid Carriers

Encapsulation is a process that needs to be carefully studied. When it is aimed to encapsulate a compound, it is important to consider the molecule structure, which is linked to its size and solubility; the characteristics of the carrier such as materials that they are made of; the application, that is, the administration route in case of clinical applications; and the material of the matrix in case of other industrial applications.

As previously mentioned, different methods and materials have been explored in order to encapsulate bioactive compounds, especially phenolic compounds, and this includes lipidic nanocarriers such as liposomes and nanoemulsions. Liposomes are vesicles composed of natural phospholipid bilayers, which allows the incorporation of lipophilic drugs in the lipid layers and hydrophilic drugs in the aqueous core [30,31]. Due to the fact that some of the phospholipids used in liposome preparation are the ones that comprise biological membranes, these carriers are commonly used as drug delivery systems for dermal applications. However, there are some drawbacks associated with the use of liposomes, including their instability, their high production cost, and the drug degradation during storage that can compromise the choice of these lipidic capsules as bioactive compound carriers [32]. Nanoemulsions consist of a system of two non-miscible liquids that form droplets at the nanometric size, with these having been used for similar goals to liposomes [33]. Usually, these systems can be oil-in-water, water-in-oil, bi-continuous, liquid-in-liquid, or liquid-in-solid nanoemulsions and the lipid phase is often made of fatty vegetable oils [34,35]. A small particle size is associated with a greater absorption and a faster release of the encapsulated compounds, but the instability of nanoemulsions that results in coalescence or flocculation phenomena is still a considerable limitation [32]. Furthermore, although a fast release might be desirable sometimes, the fact that it cannot be controlled in nanoemulsions also limits their application [36].

In contrast, and since compound mobility is usually lower in a solid state, the use of solid lipids can be the answer to accomplish a controlled drug release [11]. SLNs are made of lipids, generally in a ratio of 0.1% (*w/w*) to 30% (*w/w*), which are solid at both body and room temperatures. These lipids are dispersed in an aqueous medium and usually stabilized by a surfactant that covers the solid core, whose concentration should vary between 0.5% (*w/w*) and 5% (*w/w*) [27,37]. The lipids used in SLN preparation are usually physiological lipids that include glycerides, sterols, partial glycides, fatty acids, and waxes. All types of surfactants (neutral, ionic, and non-ionic) can be used to enhance the stability of the nanoparticles, and the combination of more than one emulsifier can help prevent particle agglomeration [11].

SLNs and NLCs have been applied in a wide variety of areas. Recent research from the last 2 years on SLN/NLC preparation include some important studies on the antiproliferative effect of cationic SLNs loaded with epigallocatechingallate (EGCG) against different cell lines [38], SLNs for the treatment of herpes simplex virus (HSV) infection [39], NLCs for antioxidant delivery [40], SLNs for food applications [41], and the enhancement of the oral bioavailability of curcumin through the use of SLNs [42]. Table 2 presents some examples of commercial lipids and surfactants used in SLN/NLC preparation, as well as some different applications.

Some advantages over other lipidic carriers that characterize SLNs are (i) the possibility of controlled drug release and drug targeting, (ii) their stability under sterilization conditions, (iii) their biodegradability, (iv) the protection of labile compounds against chemical degradation, (v) the incorporation of both hydrophilic and lipophilic drugs, and (vi) the possibility of the scaling-up process [11,43]. However, SLNs have some associated limitations such as the relatively high water content (between 70% and 99.9%), which can be a problem during incorporation into a final product, and low loading efficiency due to the tendency that lipid crystals have to recrystallize over time [44,45]. During SLN preparation, lipids crystallize in higher energy modifications, α or β’, but during storage, lipid particles can transform to more organized, low energy, β modification. This higher degree of organization will present less available space for drug loading, since compounds are often located in the imperfections of the lipid matrix, or, in other cases, between the fatty acids’ chains and between lipid layers [37].

**Figure 2:**
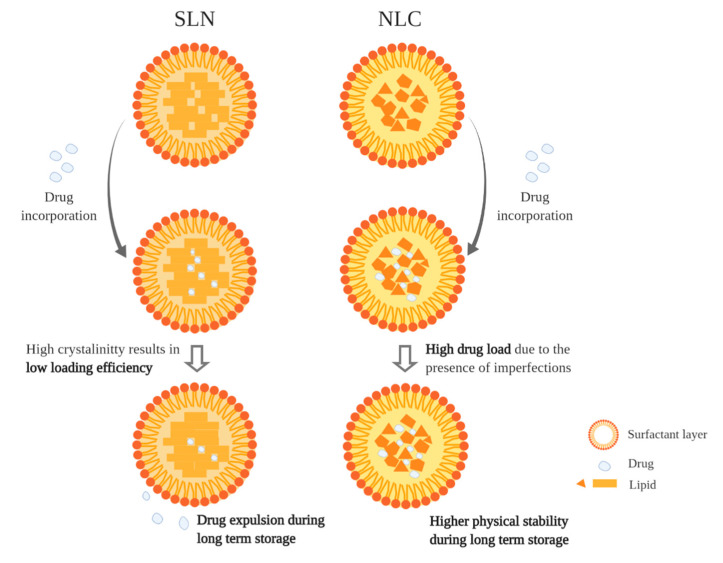
Difference between SLN and NLC loading efficiencies (adapted from [57]).

More recently, a second generation of lipid nanoparticles that include NLCs were developed to overcome some drawbacks of SLNs. NLCs are prepared using a blend of solid lipids and liquid lipids in a ratio of 70:30 to 99.9:0.1. The use of liquid lipids creates a depression on the melting point of the system and will be responsible for some enhanced properties such as minimization of drug expulsion during storage and increased loading capacity [37]. Since NLCs have liquid lipids in their constitution, the organization degree of the lipid matrix is lower, which creates more imperfections where the molecules can place themselves, as is schematized in Figure 2 [37]. On the other hand, it is important to emphasize that the localization of the drug in the SLN/NLC also depends on lipophilicity and on the structure of the target molecule [57].

**Figure 3:**
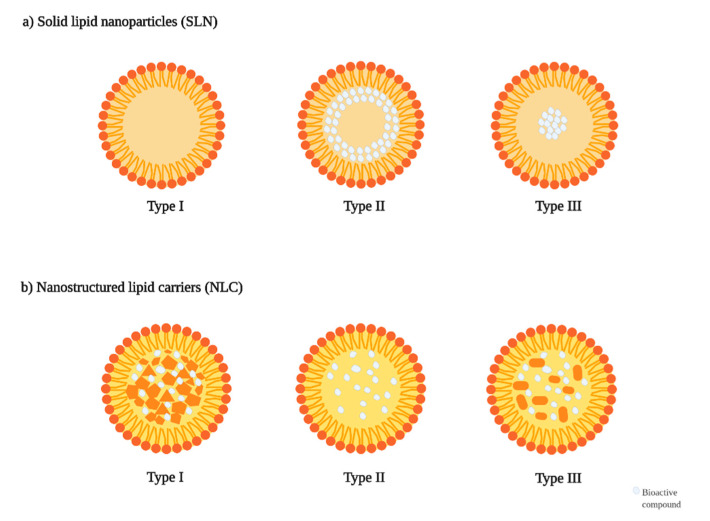
Different types of SLNs and NLCs. Type I SLN does not present any “drug” icon due to the homogeneity of the matrix; (**a**) Solid lipid nanoparticles (SLN); (**b**) Nanostructured lipid carriers (NLC).

SLNs have been differentiated among themselves as well as NLCs. For instance, in SLN type I, the homogeneous matrix model, the drug is dispersed in the lipid core while in SLN type II, the drug-enriched model, a drug-free lipid core is formed, and an exterior solid shell with both lipid and drug is formed. Both types of SLNs can be prepared with the high-pressure homogenization (HPH) method that will be further explored. On the other hand, SLN type III, the drug-enriched core model, is obtained when the drug concentration is close to its saturation solubility in the lipid, which will yield to its precipitation in the core and to the formation of a lipid coverage (Figure 3a). It is noteworthy that small changes in some relevant variables influence SLN and NLC structure and therefore their applicability. For instance, SLN type I can show controlled release properties, whereas SLN type III is adequate in achieving a prolonged drug release, while SLN type II is not suitable for this purpose [58,59].

In addition, NLC type I, the imperfect crystal model, is obtained through the mixture of sufficient amounts of liquid lipids and solid lipids, creating a matrix with a great number of voids and imperfections where the drug can place itself. NLC type II, the amorphous model, is obtained when using special lipids that do not recrystallize after homogenization and cooling, creating an amorphous lipid matrix that minimizes drug expulsion. NLC type III, the multiple model, is composed of small oil nanoparticles that are inside the solid lipid matrix due to a phase separation, and it is obtained when mixing solid lipids with higher amounts of oils in a ratio where the solubility of the oil in the solid lipid is exceeded (Figure 3b). These multiple models of NLCs are adequate to obtain a controlled drug release and enhance the loading capacity of drugs that are usually more soluble in liquid lipids [37,58,60,61].

As the interest in phenolic compounds and their beneficial health properties increases, more studies aim to explore new ways and methods to deliver these molecules to target cells or tissues. Several encapsulation methods have been described as successful carriers of such molecules for oral delivery, topical applications, and other applications in the food industry. As explained before, encapsulation of phenolic compounds represents many advantages over the application of free phenolic compounds by protecting molecules from external factors and enhancing their bioavailability or specificity. Moreover, the incorporation of these metabolites in nanocarriers provides an increased surface area and, therefore, an easier pass of the system through cell membranes [10]. The applied methods of encapsulation of phenolic compounds include spray drying, coacervation, liposomes, inclusion, co-crystallization, yeast encapsulation, emulsion, and nanoparticles [62,63,64]. Among all procedures, lipid nanoparticles have gained growing attention and have been playing a major role in phenolic compound encapsulation since a great number of these compounds are hydrophobic, with lipids being commonly found in nature and having low toxicity, and the resulting nanocarriers having a similar constitution with biological membranes. SLNs and NLCs have been demonstrated as suitable candidates for the encapsulation of phenolics due to the properties already referred [65]. The solid lipid matrix has shown to protect the chemical labile compounds from degradation, in which phenolic compounds are included [10,66].

An important issue to consider while encapsulating phenolic compounds in lipid carriers is the solubility of the molecule in lipidic matrices. Quercetin has showed good solubility in oil phases but formulation studies have demonstrated that this phenolic compound distributes itself at the oil/tensioactive interface, thus exhibiting an amphiphilic behavior [32,67]. On the other hand, it is also possible to encapsulate hydrophilic phenolic compounds, as will be explored further ahead. Phenolic compounds can also be responsible for modifications in size, shape, and surface of the nanoparticles, therefore potentially influencing their stability [10,68].

Since the aim of lipid nanoparticles is the further incorporation in cosmetic or pharmaceutical products, there are some aspects that must be considered such as the regulatory status and toxicity of its components. The introduction of a product in the market requires the use of constituents with accepted status by the regulatory authority of generally recognized as safe (GRAS) and, in general, a wide range of lipids meet this criteria and can be used to prepare lipid nanoparticles [57]. A broad range of surfactants and polymeric stabilizers are also widely used with no associated problems. However, the acceptance of materials containing nanoparticles always demands special attention, and toxicity studies are always necessary since in the nano-range, physicochemical properties such as adhesiveness or melting points are different. Thus, it is important that studies involving the development or preparation of SLNs/NLCs always include cell viability assays, which are commonly performed using the MTT (3-(4,5-dimethylthiazol-2-yl)-2,5-diphenyltetrazolium bromide) assay. An important factor to consider, as cell studies are performed in buffers or cell cultures, is that SLNs/NLCs are formulated in aqueous dispersion, and a higher concentration of electrolytes might cause precipitation [69,70]. No relevant association between the kind of lipid and observed toxicity was found since it usually also depends on the cell line and in nanoparticle structural characteristics. On the other hand, it is proposed that single alkyl chain-containing surfactants are more toxic than double-chained surfactants [71]. No significant difference between NLC and SLN toxicity was verified and the numerous studies reported in the literature on this topic show that SLNs/NLCs are well tolerated nanocarriers [69].

## 3. Preparation Methods and Functionalization Approaches

### 3.1. Preparation Methods

**Scheme 1:**
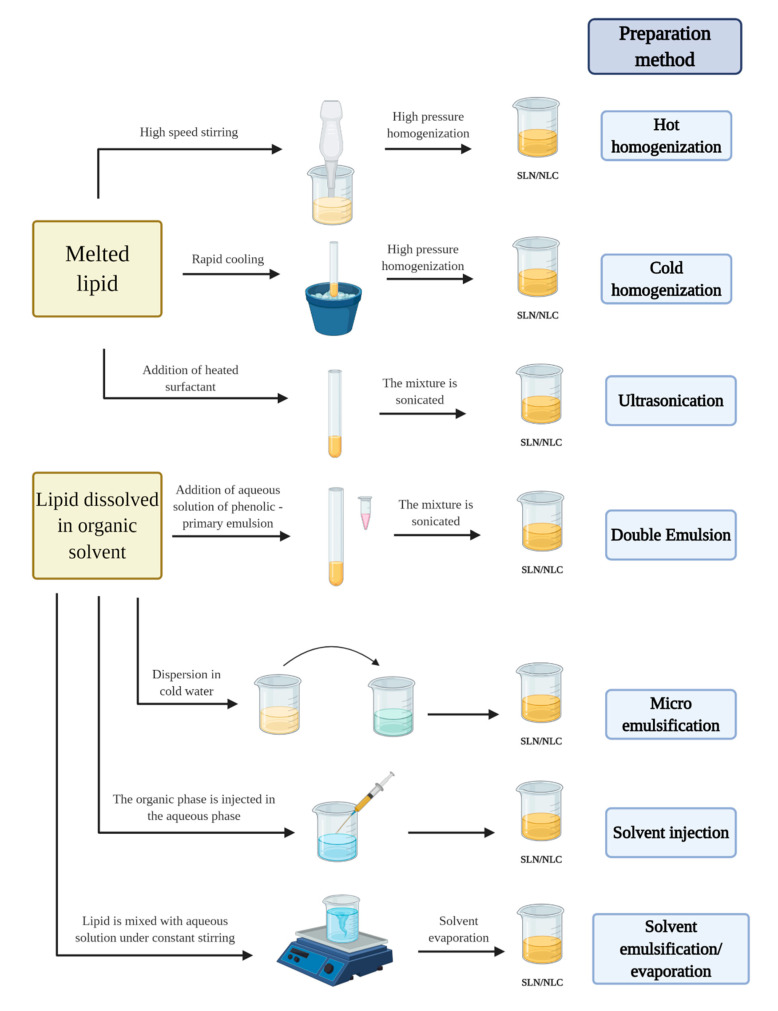
SLN/NLC preparation methods.

Several methods have been described for the preparation of SLNs/NLCs. Generally, all methods are efficient and are chosen according to the compound to be encapsulated and the final application or administration route. In this section, we highlight some well-known methods applied for the preparation of SLNs and the encapsulation of phenolic compounds that include (i) high-pressure homogenization, (ii) solvent emulsification/evaporation, (iii) micro-emulsification, (iv) solvent diffusion and solvent injection, (v) ultrasonication, and (vi) double-emulsion. Some physical–chemical parameters of phenolic compound-encapsulated SLNs/NLCs obtained by these methods are summarized in Table 3 and Scheme 1.

#### 3.1.1. High Pressure Homogenization (HPH)

HPH is one of the most used methods in SLN/NLC preparation for the encapsulation of phenolic compounds. Shtay et al. prepared SLNs loaded with EGCG using a hot high-pressure homogenization method with slight modifications [72]. For this, the authors used cocoa butter, mono- and diglycerides (MDG), and sodium stearoyl-2-lactylate (SSL). An EGCG-rich extract was dissolved in water and this solution was poured into the organic phase containing the cocoa butter and MDG, previously melted at 60 °C, and mixed using a high-speed mixer. The resulting mixture was re-emulsified after the addition of the remaining water amount containing SSL at the same temperature (60 °C). The final solution was then homogenized using a high-pressure homogenizer and then cooled. In vitro release studies were carried out using simulated gastric fluid (SGF, pH 1.2) and intestinal fluid (SIF, pH 6.8) and a more prolonged release was observed in both conditions, which will possibly lead to an improved bioavailability of EGCG [72]. Similarly, Trombino et al. prepared SLNs loaded with trans-ferulic acid using a high pressure homogenization method [73]. In this study, ferulic acid was used both as a structural constituent of SLNs and as an encapsulated bioactive compound, as this technique was reported to help in the protection of encapsulated compounds in ferulic acid-based drug carriers [66,74,75]. Stearyl ferulate or stearic acid were melted in the presence of ferulic acid, and a hot solution of sodium taurocholate, butanol, and Tween 20 was added to the melted lipids. The formed microemulsion was dispersed in water (at 2 °C) under high-speed homogenization and the SLN dispersions were washed through an ultrafiltration system. Lipid peroxidation was studied in incubations containing rat microsomes and results showed that both ferulic acid SLNs and ferulic acid SLNs formulated with stearyl ferulate counteracted lipid peroxidation induced by different oxidants, which allows for the possible application of the prepared dispersions in parenteral or oral routes for antioxidant applications [73]. Huang et al. prepared quercetin and linseed oil-co-loaded NLCs using a combination of the high-pressure homogenization technique and a melting emulsification [76]. Briefly, the lipid and surfactant were mixed and heated up to 70 °C and then quercetin and linseed oil were added to the mixture and stirred at the same temperature. Pre-heated distilled water was injected into the mixture and the resultant pre-emulsion was homogenized using a high-pressure homogenizer. The authors analyzed the characteristics of the resulting nanoparticles and also performed in vitro drug release studies and an accelerated oxidation experiment to understand the susceptibility of linseed oil to oxidation when encapsulated into NLCs, with the results showing the potential of quercetin NLCs as carriers for food applications [76].

Hot and cold high-pressure homogenization methods are both simple and cheap [60,77,78,79,80]. In general, they are largely used due to their simplicity, delivering good results in various types of drugs. However, the energy consumption and the inability to use these methods at an industrial scale are two major drawbacks. Such disadvantages are not present in other methods such as solvent emulsification/evaporation and micro-emulsification.

#### 3.1.2. Solvent Emulsification/Evaporation

Santonocito et al. prepared functionalized curcumin-loaded SLNs by solvent emulsification/evaporation technique for the systemic administration of curcumin [81]. Curcumin, Compritol 888 ATO and injectable soy lecithin were solubilized and melted in ethanol at 70 °C. The melted lipid phase was dispersed in the hot surfactant solution (Lutrol F68) under stirring and then this solution was cooled in an ice bath and the organic solvent was removed by vacuum [81]. Small particles sizes can be obtained using this methodology due to the good solubilization of the lipid in the organic solvent, with the absence of any thermal stress or heat being a great advantage towards HPH. A major fact that also deserves attention is that this method is easy to scale up and is a continuous process. Overall, this is one of the simplest methods used to obtain SLNs and does not require a deeper optimization. However, in some cases, biomolecules could get damaged during the process and the use of organic solvents are some drawbacks associated to this technique [11,82]. Such limitations are not found in the micro-emulsion technique.

#### 3.1.3. Micro-emulsion

The micro-emulsion method was used for the preparation of ferulic acid-loaded SLNs [83]. Picone et al. prepared ferulic acid-loaded SLNs by micro-emulsification technique for the delivery of this hydroxycinnamic acid [83]. The lipid phase, constituted by Compritol 888 ATO, was heated above its melting point and an aqueous phase of the surfactant, Epikuron 200, and an aqueous phase of taurocholate sodium salt were added after the dissolution of ferulic acid in the melted lipid. SLNs were obtained after dispersing the previous o/w microemulsion in cold water under mechanical stirring [83]. Similarly, Nayak et al. prepared NLCs loaded with curcuminoids by a nanoemulsion technique [84]. The aqueous phase, consisting of Poloxamer 188 and Tween 80, and the organic phase, consisting of a mixture of solid and liquid lipids, curcuminoids, and soybean PC (phosphatidylcholine), were separately heated up to 75 °C. The lipid phase was dispersed in the aqueous phase and mixed by high shear homogenization and the nanoemulsion was further obtained by ultrasonication following by a dispersion in cold water containing polyvinyl alcohol (PVA) at 2 °C under high-speed homogenization. Physicochemical characteristics were evaluated as well as the in vitro release profile and the in vivo antimalarial activity. The results suggest that the developed formulations can be administrated parenterally, hich can help to obtain higher concentrations of bioactive compounds in the liver, spleen, and blood, which are the sites where parasites are usually localized [84].

This method might be the most appropriate when the aim is to encapsulate phenolics that are more sensitive to mechanical stress, however, the resulted preparation is usually very sensitive to change [60]. Other methods known for their reduced shear stress and low energy input are the solvent diffusion and solvent injection methods.

#### 3.1.4. Solvent Diffusion and Solvent Injection

Pandita et al. prepared resveratrol-loaded SLNs using the *solvent diffusion* method [85]. Stearic acid and Phospholipon 90 G were dissolved in ethyl acetate and this solution was injected through a syringe to an aqueous solution containing poloxamer 188 as a surfactant. The results showed that encapsulation protected resveratrol from photodegradation and in vitro drug release studies revealed a sustained drug release from the resveratrol SLNs; moreover, pharmacokinetic studies performed in male Wistar rats showed an 8.035-fold improvement in the oral bioavailability of resveratrol after oral administration of resveratrol SLNs when compared to its pure suspension. Altogether, this study shows that problems related to low bioavailability of resveratrol after oral administration can be overcome by the encapsulation of the molecule into SLNs [85]. The main advantages of these methods include the small particle size obtained and the avoidance of heat during the procedure. However, the possibility of having residual solvent in the final product is a drawback [60,86,87]. Such problems are not found in methodologies where the lipid is melted rather than dissolved in an organic solvent.

#### 3.1.5. Ultrasonication

Han et al. prepared quercetin-loaded SLNs by a homogenization/ultrasonication method [28], wherein the lipid phase, containing the lipid and drug or bioactive compound, and the aqueous phase (surfactant) are separately heated at approximately 5 °C above the lipid melting point. The aqueous phase is then added to the lipid phase and this mixture is homogenized extensively using a probe-sonicator [88]. This is a fast and simple procedure that requires a small number of reagents and equipment. The major drawback can be associated with poor lipid dissolution and consequently bigger particle sizes, especially with more hydrophilic molecules. Such a limitation can be overcome by the use of methods developed especially for the encapsulation of these molecules such as the double-emulsion method.

#### 3.1.6. Double-emulsion

Since the lipid matrix of SLNs/NLCs is hydrophobic, some highly hydrophilic drugs are expected to be weakly encapsulated. The double-emulsion method, usually applied in the preparation of polymeric nanoparticles, seems to be a promising way of solving this inconvenience [89]. In this method, the hydrophilic compounds are first dissolved in water while lipids are dissolved in an organic solvent [90]. The water phase is dispersed in the organic solvents and a primary emulsion is formed (*w*/*o*). Then, this emulsion is dispersed in an external water phase (surfactant) to form a double emulsion water-oil-water (*w*/*o*/*w*). The organic solvent is evaporated in the end. Ravanfar et al., using a double emulsion technique, prepared SLNs loaded with anthocyanins to improve the chromatic stability towards pH change and high temperatures and, as was expected, the encapsulation of anthocyanins was a challenge since these molecules have a tendency to partition into aqueous phase during preparation [91]. The lipid phase, containing palmitic acid, Span 85, and egg lecithin was heated at 60 °C and ethanol or isobutanol were added as co-surfactants. The *w*/*o* microemulsion was obtained by titrating the lipid phase with a solution containing anthocyanins from red cabbage in distilled water under stirring, with the final solution being dispersed in an aqueous solution of Pluronic F127 under high shear homogenization [91].

Although there are many advantages associated with these lipid nanocarriers, there are a few limitations, especially those related to preparation and storage, that must be considered when optimizing a process. These problems are usually related with drug expulsion during storage, increases in particle size, the gelation phenomenon, the occurrence of supercooled melts, the particle shape change since lipids tend to crystallize in the platelet form, or the already mentioned lipid modifications and the coexistence of other colloidal species [11,92].

The applied method during nanoparticle preparation can have a significative relationship with the stability of nanoparticles during storage. Sometimes, the high-pressure homogenization method can induce drug degradation due to high shear stress. This was observed in the encapsulation of DNA and albumin using this procedure [93]. There are also surface-related phenomena and lipid-surfactant interactions that may dictate the possibility of lipid crystallization. Among various factors, the existence of lipid modifications and the gelation phenomenon are of special importance.

Lipid modifications have higher mobility in thermodynamically unstable configurations; therefore, these configurations have a greater capability of drug incorporation. However, during storage, in favor of thermodynamically stable configurations, rearrangement of crystal lattice is likely to occur in what might result in drug expulsion [11]. Nevertheless, an in vitro study on skin showed, the evaporation of water leads to lipid modifications in SLN dispersions that result in drug expulsion and increased penetration into the skin, which was a beneficial aspect in this situation [94].

The gelation phenomenon is related to the transformation of the SLN dispersion into a viscous gel and it is mostly associated with intense contact with other surfaces and shear forces, being accelerated with increasing storage temperature and increasing light exposure [95]. This process is irreversible and some authors have shown that crystallization phenomena, high lipid concentrations, high ionic strength, and the performance of the surfactant film (that might change with temperature, especially in polyethylene glycol (PEG) surfactants) are factors that might be at the origin of gelation. The zeta potential is usually a good indicator of the probability of occurrence of the gelation phenomena since values close to −15 mV could be a sign of the future formation of a viscous gel [70,96].

Moreover, drug release is one of the key topics to be explored when studying lipid nanocarriers, and NMR techniques are particularly useful to study the molecular rearrangement of the drug and the lipid [11]. Drug release can be fast, sustained, or controlled in several ways according to the final application. This parameter is intrinsically related with loading capacity since it will mainly depend on the localization of the compound in the nanoparticle. For instance, if the drug has affinity for the lipid and also localizes itself in the core of the nanocarrier, then it is expectably a prolonged drug release whereas when the drug has a complex and wide structure and localizes itself at the surface of the nanoparticle it is expectably a fast drug release. Prolonged drug release can be useful when developing products such as cosmetics, whereas a burst effect on the release of the compound is desirable when the main goal is to have an immediate effect, for example, in pain relief medicines.

The extent of burst release is also influenced by other factors and methods. For example, nanocarriers are frequently modified, namely, at the surface, to have a selective and targeting behavior, consequently presenting different release profiles since in some cases the release of the drug is activated by the presence of a specific molecule or a specific pH value [97]. More commonly, the burst release effect is controlled by regulating the drug solubility in the aqueous phase of the SLN/NLC preparation, which depends on the chosen temperature and surfactant concentration. When higher temperatures and concentrations are applied, the burst effect increases. The use of surfactants unable to solubilize the drug normally decreases the burst effect and is, therefore, more adequate in terms of achieving a prolonged release [26,98].

To assess drug release profiles in vitro, the methods generally used include dialysis and the static or dynamic Franz diffusion that can be designed in order to mimic the administration route, for example, adjusting pH and temperature [43]. In the dialysis method, SLN/NLC dispersions are poured into a dialysis bag that is placed in a dissolution media (that can be a buffer at a controlled temperature) under stirring. A fixed quantity of samples is withdrawn at fixed time intervals and the concentration of drug in the samples is assessed using spectrophotometric or chromatographic methods [88].

### 3.2. Functionalization Approaches

**Figure 4:**
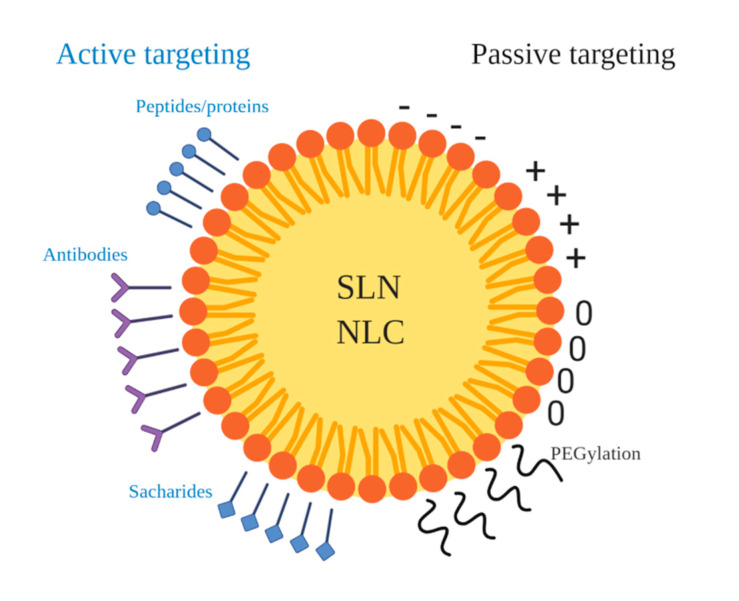
Examples of active and passive targeting for the functionalization of SLNs/NLCs (adapted from [101]).

A common requirement for the appropriate action of nanoparticles is a controlled interaction with biomacromolecules [99]. Drug targeting is a challenging task when developing carriers for drug transport/delivery. Generally, the human body identifies hydrophobic molecules as foreign and for that reason hydrophobic nanoparticles are readily taken up by macrophages [100]. For this reason, when the goal is the retaining of nanoparticles in blood circulation, additional measures have to be taken in order to prevent nanoparticles phagocytosis. Surface modification can be used not only to protect nanoparticles from macrophages, but also to enhance cellular internalization; provide selective recognition and binding; improve binding capacity, essential to efficient intracellular delivery; increase blood circulation time; and, consequently, improve drug efficacy and allow for the reduction of the administered drug dose [101,102,103,104]. The functionalization of nanoparticles can be achieved using different strategies, namely, the coating of the nanoparticle with ligands such as surfactants, small molecules, polymers, and biomolecules (Figure 4) [103]. While planning the functionalization of nanoparticles, it is important to consider different factors such as (i) particle size, (ii) surface charge, (iii) hydrophobicity, (iv) target cells, and (v) administration route among other relevant parameters. As an example, it is known that positively charged nanoparticles are preferentially taken up by cells and by this, coating of nanoparticles with molecules with positive charge has become a commonly used strategy to enhance cellular uptake [101,105,106,107]. As referenced to before, hydrophobicity is especially important when using an intravenous administration route due to opsonization of nanoparticles by macrophages. In this case, it is typical to functionalize nanoparticles with hydrophilic polymers and surfactants (e.g., polysorbate 80, poloxamers, PEGs) in order to enhance nanoparticle bloodstream circulation [100,101,108,109,110,111]. Surface functionalization can be done with active targeting in mind, including the functionalization of the nanoparticle with specific ligands to directly bind to or interact with specific cell sites or a passive targeting that is related to the ability of nanoparticles to reach the target cells on the basis of their physicochemical characteristics [101,112]. This last type of targeting is related to the enhancement of the retention effect that is based on the fact that some cells have the ability to better concentrate or retain nanoparticles in comparison to other cells or tissues [112].

One of the most important forms of passive targeting is based on the conjugation of polyethylene glycol (PEG) chains to the surface of nanoparticles, known as PEGylation [113]. PEGylation is extensively used to protect nanoparticles from opsonization and to inhibit connections with serum components since PEG chains decrease protein adsorption on the nanoparticle surface [101]. Moreover, PEG chains are cleaved by matrix metalloproteinases, which allows for their efficient application in tumor cells where the concentration of these proteinases is increased in surrounding areas [114]. Several studies have even taken advantage on the functionalization with PEG chains and have combined it with other targeting molecules such as carboxylic acids or amines present in different targeting ligands [81,101,108,115,116].

Active targeting is more selective because it relies on the binding of specific molecules to specific ligands of the target cells or tissues through highly specific interactions [101,117]. Active targeting includes surface functionalization with antibodies, saccharide ligands, and other biomolecules and is particularly important in cancer therapy studies since it confers specificity to the nanoparticle to only target cancer cells. A widely explored functionalization type is antibody functionalization. The modification of nanoparticle surface with antibodies can be performed directly or through linker ligands such as PEG [101,118,119].

The use of other biological molecules such as proteins for functionalization have also been explored. Lipoproteins enter the blood-brain barrier endothelium through endocytosis via low-density lipoprotein (LDL) receptors and are transcytosed to the brain [120]. Neves et al. prepared resveratrol-loaded SLNs with cetyl palmitate as lipid phase and polysorbate 80 as surfactant and functionalized them with apolipoprotein E (Apo-E) in order to mimic lipoproteins [121]. The functionalization was performed by addition of avidin onto the surface of SLNs, allowing the non-covalent interaction between avidin and biotinylated Apo-E. The results showed a significant increase of resveratrol permeability in the blood-brain barrier (BBB) when encapsulated in functionalized SLNs, suggesting that these nanosystems can be used for the efficient brain delivery of resveratrol [121].

## 4. Applications of SLNs/NLCs Loaded with Phenolic Compounds

Due to their characteristics, phenolic compounds are applied in varied areas. Various studies aim to enhance their efficacy through the use of SLNs/NLCs as carrier systems to prepare cosmetic and pharmaceutical products or functional foods [7,73,122].

### 4.1. Cancer Applications

Curcumin is a lipophilic phenolic compound whose anti-cancer effects have been widely explored in recent times [123]. This phenolic molecule reduces cell proliferation and metastasis and accelerates apoptosis, and for this reason, has been indicated as a good candidate for treatment and prevention of breast cancer [123,124,125,126]. Similar to other phenolic compounds, curcumin presents some associated limitations in oral administration since it is poorly aqueous, soluble, and easily degrades itself. Baek et al. took advantage of SLN technology to produce a delivery system that improves the stability of curcumin [123]. Different SLNs loaded with curcumin were prepared using glyceryl monostearate (GMS), soya lecithin, stearylamine, and poloxamer 188 as surfactant. Moreover, the surface of the nanoparticles was modified with N-carboxymethyl chitosan (NCC) in order to enhance biocompatibility and absorption of the nanoparticles. Studies performed in the MCF-7 cell line (breast cancer cell line) revealed that encapsulation of curcumin improved their cellular uptake. Furthermore, in vivo experiments involving rats showed that oral bioavailability of curcumin was improved and the burst release of the compound in gastric medium was prevented when using surface-modified SLNs. The curcumin uptake at the intestinal lymphatic node was also enhanced. Altogether, these results present curcumin-loaded SLNs (and surface modification with chitosan) as a promising way to increase oral bioavailability and antitumor effects of this phenolic compound, opening doors for the possible development of pharmaceutic formulations for the treatment of breast cancer.

EGCG, commonly found in green tea, has presented, equally to other phenolic compounds, several beneficial health effects, namely, anti-carcinogenic properties [127,128,129]. Silva et al. evaluated the anti-proliferative effect of EGCG-loaded SLNs against several cell lines. SLNs were prepared with glycerol, Softisan 100, Lipoid S75, dimethyldioctadecylammonium bromide (DDAB), and poloxamer 188 as surfactant [38]. The results showed that EGCG exhibited anti-proliferative effects against different cell lines in the following order of potency: MCF-7 > SV-80 > HepG2 > Y-79 > Caco-2. These results were obtained after 24 h of exposure and clearly show that the anti-proliferative effect of the phenolic compound is time-, concentration-, and cell-line-dependent [38].

Resveratrol has also been studied for its possible application as an anti-cancer agent since it is known to target many components of intracellular signaling pathways [130]. However, the beneficial effects of this molecule are compromised by its low water solubility and photo instability [131]. Teskač et al. prepared SLNs with Compritol 888 ATO, Phospholipon 80H, and Lutrol loaded with resveratrol to investigate the cell uptake, transport, and internalization of this phenolic compound in keratinocytes (NCTC2544 cell line) [132]. The results showed that the delivery of resveratrol by SLNs contributed to the effectiveness of the molecule on decreasing cell proliferation, anticipating its possible formulation for skin cancer treatment.

It is also common to encapsulate more than one phenolic compound in order to achieve an enhanced final effect. Kumar et al. prepared functionalized resveratrol and ferulic acid-loaded SLNs with the aim of developing cell-targeted drug delivery systems. The co-encapsulation of both phenolic molecules has the goal of combining the beneficial properties of resveratrol, known for its beneficial effects such as cardioprotection and cancer prevention, and ferulic acid, known to inhibiting colon cancer cells [133,134]. It is important to note that the characteristics of the nanoparticles are usually not altered by the co-encapsulation of two phenolics [135]. The authors investigated the physicochemical properties of the nanoparticles, drug release, and the anti-cancer effects studied in colon cancer cells (HT-29) and results indicated that the prepared SLNs were cancer- cell-specific and induced cell death in HT-29 cell lines [136].

### 4.2. Oral Bioavailability

As previously mentioned, phenolic compounds are present in a wide variety of vegetables and fruits and are, therefore, a fundamental part our diet. However, their biological effects are compromised by their instability and/or low bioavailability. Thus, many studies are aiming to encapsulate phenolic compounds to enhance their bioavailability after food consumption, which can be of great use in the development of functional foods. Granja et al. developed NLCs loaded with EGCG and functionalized with folic acid, with the aim of improving their oral absorption. NLCs were prepared with Precirol ATO 5 and Mygliol 812 with Tween 60 as a surfactant and were functionalized with folic acid [137]. The results showed a controlled release of EGCG in simulated gastrointestinal conditions and a high stability up to 8 weeks (4 °C, protected from light). Such data shows the suitability of SLNs for the preparation of formulation containing EGCG for the development of products for the food industry.

### 4.3. Skin Applications

Cosmetics is an area where lipid nanoparticles stand out and find different putative applications. The similarity between the lipid matrix of these particles and the skin lipids, the capacity of improving skin hydration resulting in enhanced skin elasticity, and the possibility of increasing the stability of natural compounds, explain why solid lipid nanoparticles are such promising nanocarriers for topical applications. Likewise, phenolic compound attributes make them good candidates for skin treatments or other applications.

More recently, lipid nanoparticles have been used as carriers of phenolic compounds for developing sunscreen formulations. These formulations have less noxious effects than those that usually contain synthetic agents [122]. Bose et al. prepared different quercetin-loaded SLNs that showed that higher amounts of quercetin were found to be localized within the skin compared to a control formulation containing particles in the micrometer range during an in vitro skin permeation study [138]. Quercetin had already been pointed as an inhibitor of Ultraviolet light B (UVB)-induced oxidative skin damage, which increases their potential application in sunscreen protector formulations [139]. In another study, Plianbanchang et al. described the efficacy of a facial cream containing curcuminoid-loaded SLNs in healthy volunteers that showed decrease in skin wrinkle formation and improved skin hydration when compared with free curcuminoids [140].

In another study performed by Friedrich et al., the authors prepared NLCs loaded with both curcumin and resveratrol to study their behavior upon application onto excised human skin and their interaction with human primary skin cells. Skin cells treated with NLCs for 24 h showed great potential of these carriers for further investigations on the subcellular level. Skin penetration studies showed an increased delivery of resveratrol (upon co-delivery) into deeper skin layers when compared to encapsulation of only resveratrol itself. These results portray the prepared lipid nanocarriers as good curcumin/resveratrol delivery systems for skin applications [141].

### 4.4. Neurological Diseases

Antioxidant compounds have a key role in protecting cells from oxidative injuries that are at the origin of several diseases such as Alzheimer’s disease. Alzheimer’s disease is a neurodegenerative disorder where there is deposition of amyloid β-peptide (Aβ) and the intraneuronal accumulation of neurofibrillary tangles, which is all associated with the loss of neurons [142]. The long-term administration of ferulic acid in mice has been shown to protect them against learning and memory deficits that are Aβ-induced [143]. Bondi et al. prepared SLNs and NLCs loaded with ferulic acid and investigated the ability of the nanoparticles to penetrate human neuroblastoma cells (LAN 5 cell line) [144]. Different formulations of SLNs and NLCs were prepared using Compritol 888 ATO, Compritol HD5 ATO, Miglyol 812, and Pluronic F68 as surfactant. The final data showed that ferulic acid-loaded SLNs had higher protective activity than free ferulic acid against oxidative stress induced in neurons, supporting the potential use of these nanocarriers to deliver ferulic acid for the treatment of Alzheimer’s disease.

## 5. Conclusions and Current Challenges

Phenolic compounds have gained growing attention due to their chemical properties and health benefits. The diversity of these molecules allied to the fact they have natural origin provides the opportunity to apply them in a wide variety of areas, namely, in the development of nutraceuticals, cosmetics, and functional foods. However, some drawbacks associated with low bioavailability and instability are blocking its application in such fields. Numerous efforts have been performed to overcome these limitations. The most common strategies include molecule covalent modification or encapsulation. Encapsulation of phenolic compounds is already widely explored but it is clear that there is some lack of information about the application of encapsulated polyphenols in the market. This is probably due to the uncertainty of some results, which further studies and exploration, or more commonly to the difficult scaling up of the processes. This review demonstrates the suitableness of lipid nanocarriers, namely, SLNs and NLCs, for the encapsulation and delivery of phenolic compounds for different applications. These lipid nanocarriers meet several desired criteria for nanosystems since they present exceptionally low toxicity, have a notorious resemblance with biological systems, and have preparation methods that are easy to scale up. However, further studies on the interaction of phenolics with lipids from SLNs/NLCs, as well as on the interaction of these molecules with surfactants and on the mechanisms of transport and cellular uptake, are necessary to achieve optimum conditions. Another important topic that needs to be explored is the fact that although the structural characteristics of lipid nanoparticles allow them to be appropriate drug carriers for lipophilic compounds, it is common to have some additional problems when it comes to the encapsulation of hydrophilic compounds. There are some essential physicochemical concepts to attend to while encapsulating hydrophilic compounds such as the solubility of the molecule, the partitioning, and the mass transport phenomena [145]. For instance, hydrophilic phenolics have many hydroxyl groups that may interact with the polar head of some lipid molecules and create complexes that can result in aggregation and/or increases in particle size [10,68]. The solubility of the bioactive compound or drug must be always analysed not only in the different phases of the delivery system but also in the surrounding application matrix. In the particular case of hydrophilic molecules, the equilibrium of water solubility of the compound determines the maximum amount that can be dissolved in aqueous solution; usually, the ionized form of the molecule has a higher water solubility than the neutral form [145]. Regarding mass transport phenomena, it is also crucial to consider that the movement of molecules from one region to another of the colloidal system will impact its loading and its release [146]. As demonstrated above, some studies have already been reported to successfully encapsulate hydrophilic polyphenols such as EGCG in SLNs/NLCs [137,147]. Other studies have also managed to encapsulate anthocyanins, which are hydrophilic pigments, in nanoemulsions or nanoliposomes, with a study of Ravanfar et al. (2016) involving the preparation of SLNs loaded with anthocyanins for the preservation of these molecules against factors such as pH and temperature [91,148,149]. Encapsulation of anthocyanins in lipid nanocarriers is a poorly explored theme that is gaining attention since these natural molecules comprise a great feature of beneficial health effects and present vibrant and attractive colours. However, anthocyanins are also characterized for their instability towards pH changes and other external factors, and their varied structures makes the encapsulation of these phenolics a particular challenge. Thus, incorporation of anthocyanins in lipid nanocarriers is a key topic that is open to deeper exploration.

## Figures and Tables

**Table 1:** Examples of phenolic compounds, food sources, applications, and beneficial effects.

Main Class	Subclass	Phenolic Compound	Food Sources	Beneficial Effects	Possible Applications
Flavonoids	Flavan-3-ol	Epigallocatechin gallate	Green tea	Anti-oxidant effects [12]	Protection against chemical carcinogens [13]
	Flavones	Apigenin	Oranges	Inhibition of rat brain monoamine oxidases (MAOs) [14]	Development of antidepressant and antianxiety agents [15]
	Flavonols	Quercetin	Onions	Anti-oxidant effects and anti-inflammatory effects [16]	Neuropathology prevention [17]
	Anthocyanins	Delphinidin-3-glucoside	Grapes	Anti-oxidant and anti-carcinogenic effects [18]	Inhibition of breast cancer cell proliferation [19]
	Flavanones	Hesperetin	Citrus fruits	Activity against apoptotic neurodegeneration [20]	Development of therapeutic agents for Alzheimer’s disease [20]
	Isoflavones	Glycitein	Soybean	Estrogenic effects [21]	Several health effects and disease prevention [21]
Phenolic Acids	Hydroxybenzoic acids	Gallic acid	Green tea	Anti-inflammatory activity [22]	Development of novel and organic anti-inflammatory agents [22]
	Hydroxycinnamic acids	Caffeic acid	Wheat, rice, and oats	Anti-bacterial activity [23]	Development of alternatives to overcome antibiotic resistance [23]
Stilbenes		Resveratrol	Grapes	Anti-oxidant effects [24]	Skin anti-aging products
Lignins		Lignin	Plants (secondary cell walls)	Development of nanoparticles for drug delivery [25]	Cancer therapy

**Table 2:** Examples of solid lipid nanoparticle (SLN) and nanostructured lipid carrier (NLC) applications, lipids, and surfactants used in their preparation.

Lipid Nanoparticle	Incorporated Molecule	Lipids	Surfactants	Application
SLN	Vitamin E	Precirol ATO 5	Tween 80	Skin applications [46]
SLN	Quercetin	Glyceryl monostearate (GMS)	Tween 80 and polyethylene glycol (PEG) 400	Food applications [47]
SLN	Baicalen	Tripalmitin, Gelucire 48/9, and Gelucire 62/5	Poloxamer 188	Treatment of ischemic stroke [48]
SLN	Apomorphine hydrochloride	Tripalmitin, hydrogenated soybean phosphatidylcholine (HSPC), glyceryl monostearate (GMS), and polyethylene glycol monostearate (PMS)	Pluronic F68 L-ascorbic acid	Treatment of Parkinson’s disease [49]
SLN	Tetandrine	Compritol 888 ATO	Myrj 52	Treatment of ocular diseases [50]
SLN	Ofloxacin	Palmitic acid	Polyvinyl alcohol (PVA)	Improvement of pharmacological activity [51]
NLC	Curcumin	Precirol ATO 5 and Miglyol 812	Lutrol F68 and Tween 80	Intraperitoneal administration [52]
NLC	Calcipotriol, methotrexate	Precirol ATO 5, squalene mixture, Myverol 18-04K	Pluronic F68	Treatment of psoriasis [53]
NLC	Paclitaxel	Glyceryl monostearate (GMS), soya lecithin, soybean oil	Hexadecyl trimethyl ammonium bromide (CTAB)	Cancer targeting [54]
NLC	mTHPC (commercial formulation Foscan)	Soybean oil and Suppocire NB	Lipoid S75, Myrj S40	Photodynamic therapy [55]
NLC	Mediterranean essential oils	Labrafil, Softisan 100	Kolliphor RH40, Tween 80	Treatment of *Candida* skin infections [56]

**Table 3:** Physical-chemical properties of phenolic compounds encapsulated in SLNs using different methods. n.d, non determined

Phenolic Compound	Preparation Method	Size (nm)	Polidispersity Index (PdI)	Zeta Potential (mV)	Encapsulation Efficiency (EE) or Loading Capacity (LC) (%)
Epigallocatechin gallate	Hot homogenization	107–122	0.102–0.149	−56.7 to −52.9	EE 50–70
Curcumin	Solvent emulsification/ evaporation	130–180	0.240–0.260	−31 to −24 mV	EE 82
Ferulic acid	Micro-emulsification	96.16 ± 2.96	0.196 ± 0.038	Water: −36.40 ± 1.38 NaCl 0.9%: −1.97 ± 0.25 PBS: −11.7 ± 2.24	LC 20
Resveratrol	Solvent diffusion–solvent evaporation	134 ± 7.6	0.182 ± 0.017	−34.3 ± 2.5	EE 88.9 ± 3.1
Quercetin	Ultrasonication	416.9 ± 11.4 to 341.2 ± 1.0	n.d.	−50.4 ± 0.0 to 29.4 ± 0.4	EE 15.2 ± 1.1 to 46.2 ± 2.2
Polyphenol-enriched *Hibiscus sabdariffa*	Double emulsion	66.5 ± 0.3 to 294 ± 2	0.17 ± 0.02 to 0.29 ± 0.01	−29.1 ± 0.6 to −21.0 ± 1.0 mV	EE 52.9 ± 0.9 and 93 ± 3
